# Prevalence and risk factors of non-specific low back pain among amateur overhead athletes in Gauteng Province

**DOI:** 10.17159/2078-516X/2025/v37i1a18797

**Published:** 2025-05-15

**Authors:** TP Moatshe, M Dawood

**Affiliations:** Department of Physiotherapy, Sefako Makgatho Health Sciences University, Ga-Rankuwa, South Africa

**Keywords:** Lower back pain, overhead athletes, scapular dyskinesia, muscle flexibility

## Abstract

**Background:**

Non-specific low back pain (NSLBP) is prevalent among athletes engaged in repetitive overhead movements, often linked to sport-specific biomechanical demands. However, research on its prevalence and risk factors in South African amateur athletes is limited, despite the high participation in sports.

**Objectives:**

This study aimed to determine the prevalence of NSLBP and identify sport-specific risk factors among amateur overhead athletes in Gauteng Province, addressing a critical gap in the literature.

**Methods:**

This cross-sectional study involved 52 amateur overhead athletes aged 18 and above who participated in volleyball, basketball, netball, soccer goalkeeping, tennis, and swimming. Data collection included self-reported NSLBP prevalence, clinical tests for scapular dyskinesia (SD) and latissimus dorsi flexibility, and the Keele STarT Back Screening Tool. Statistical analysis examined the associations between NSLBP, SD, and sport-specific factors.

**Results:**

NSLBP prevalence was 25%, with volleyball (33%) and basketball (30%) athletes most affected. Scapular dyskinesia was observed in 48% of participants, particularly grades 1 and 2. Reduced latissimus dorsi flexibility was present in 40% of athletes and was significantly correlated with scapular dyskinesia (p<0.05).

**Conclusion:**

The study successfully met its objectives by identifying the prevalence of NSLBP and sport-specific risk factors among amateur overhead athletes. Findings emphasise the importance of targeted interventions focusing on scapular function and muscle flexibility to reduce NSLBP incidence. These insights provide valuable guidance for clinical practice and preventive strategies.

Non-specific low back pain (NSLBP) is common among athletes, particularly those participating in sports requiring repetitive overhead movements. These sports significantly strain the lower back, increasing the risk of NSLBP. Studies show that athletes in sports involving repetitive spinal loading are more likely to experience NSLBP, with prevalence estimates ranging from 20% to 30%.^[1–3]^ Despite these figures, there is limited research on the prevalence and risk factors of NSLBP among amateur overhead athletes in South Africa. Understanding these aspects is crucial for developing effective prevention and treatment strategies.^[1]^

The unique biomechanical demands of overhead sports, such as volleyball, basketball, and tennis, impose distinct stresses on the lumbar spine and surrounding muscles, potentially leading to non-specific low back pain.^[2]^ These sports require repeated overhead actions that strain the lower back and disrupt normal spinal mechanics.^[2]^ The kinetic chain concept highlights the interdependence of body segments— including the scapula, shoulder, and lower back—necessary for efficient movement.^[3]^ Disruptions in this chain, such as those caused by scapular dyskinesia, can lead to compensatory movements and increased stress on the lumbar spine.^[3]^

Scapular dyskinesia, for instance, can alter shoulder mechanics, leading to increased lumbar lordosis and subsequent strain on the lower back muscles. Research indicates poor scapular control is a significant risk factor for shoulder and lower back injuries among overhead athletes.^[4]^ This emphasises the importance of understanding how these sport-specific factors contribute to NSLBP, as dysfunction in one part of the kinetic chain can impact the entire system.^[5]^

Given this background, the objectives of this study are to determine the prevalence of NSLBP among amateur overhead athletes at a tertiary institution in Gauteng Province, South Africa, and to identify sport-specific risk factors associated with this condition. By elucidating the relationship between the mechanisms of overhead work and NSLBP, this research aims to fill a gap in the literature and provide valuable insights for clinical practice and preventive measures.

## Methods

### Study design and participants

This cross-sectional study involved 52 amateur athletes (27 males and 25 females) who participated in overhead sports at a tertiary university in Gauteng Province, South Africa. The sports included volleyball, basketball, netball, soccer goalkeeping, tennis, and swimming. Participants were recruited through university sports clubs, and notices were sent to sports teams on campus. Ethical approval for this study was obtained from the Sefako Makgatho Health Sciences University Research Ethics Committee (SMUREC/H/271/2022:PG).

### Definition of amateur athlete

For this study, an “amateur athlete” was defined as an individual who participates in sports primarily for personal enjoyment, hobbies, fitness, and competition without receiving significant monetary compensation or professional contracts.^[6]^ The inclusion criteria required participants to be 18 and above, actively involved in overhead sports registered for the academic year 2023 and classified as amateur athletes based on self-reported status and lack of professional contracts. The exclusion criteria comprised elite amateur athletes, individuals who had undergone recent surgery or experienced injuries to the shoulder or spine within the past five years, neurological diseases, congenital spinal deformities, and other medical conditions that could influence the study’s outcomes.

### Pilot study

A comprehensive pilot study with ten participants was conducted before the main study to thoroughly evaluate the feasibility and reliability of the data collection methods and tools. The objectives of the pilot study were to test the practicality of the study design, assess the clarity and effectiveness of the data collection instruments, and identify any potential issues that could affect the final study.

The pilot study involved ten overhead athletes from local netball players and were of the target population in terms of age, gender, and athletic background. This sample size was selected to provide preliminary insights without exhausting resources. The pilot study utilised the same data collection tools and procedures planned for the main study, including questionnaires, test procedures, and measurement techniques

Participants reported confusion regarding some questions, particularly those involving complex terminology or ambiguous wording. The questionnaire was revised to simplify language and clarify questions. Additional instructions were provided to ensure that participants understood each item. Pilot participants were asked to review the revised questionnaire to confirm that the changes addressed their concerns.

Detailed step-by-step procedures for the tests were developed and tested. These instructions included visual aids and examples to improve participant understanding and adherence. The revised guidelines were reviewed with pilot participants to ensure they were easily followed and reproducible. Standard operating procedures were established to ensure consistency in measurement techniques. Training sessions were conducted for all involved personnel to standardise procedures and reduce variability. Measurement tools were calibrated to ensure accuracy. The feedback from the pilot study was crucial in refining the study design. By addressing the issues identified in the pilot study, the final research was better equipped to produce accurate and reliable results, contributing to the validity of the research findings.

### Data collection

Participants completed a detailed data collection sheet that gathered information on age, gender, hand dominance, and sports participation details, including sport type, position, and training frequency. Participants also provided information about the duration and severity of lower back pain and its impact on their performance.

The Keele STarT Back Screening Tool was used to assess the participants’ risk and severity of low back pain. This tool consists of nine questions about the participants’ back pain experience over the last two weeks. The total score helps classify participants into low, medium, or high-risk categories for developing persistent disabling back pain.^[7]^

### Clinical tests

Two clinical tests were performed to assess musculoskeletal health: the Scapular Dyskinesia Test (SDT)^[8,9]^ and the Latissimus Dorsi Length Test (LDLT).^[10]^ These tests were conducted on participants already included in the study. Each test was performed twice for both arms to ensure reliability, with measurements taken by two trained physiotherapy third-year students and 1 qualified physiotherapist.

#### Scapular Dyskinesia Test (SDT)

This test involves observing scapular movement during shoulder abduction and flexion to identify any abnormal patterns indicative of dyskinesia.^[8,9]^ Scapular dyskinesia was graded as follows:

- Grade 1: The inferior angle of the scapula is more prominent.
- Grade 2: The medial border of the scapula is more prominent.
- Grade 3: The superior border of the scapula is more prominent.

Measurements were taken with and without weights to assess the consistency of dyskinesia.

#### Latissimus Dorsi Length Test (LDLT)

This test measures the flexibility of the latissimus dorsi muscle, which can impact scapular movement and contribute to NSLBP. The participant lays supine with knees flexed while the examiner (main researcher) measured active shoulder flexion with the arm in external rotation. Any lumbar extension or anterior pelvic tilt observed during the test indicated a short latissimus dorsi muscle. Reduced flexibility was defined as an angle of less than 180 degrees. While previous studies have used passive measurement techniques,^[10,11]^ the present study used active shoulder flexion, allowing for functional assessment of latissimus dorsi flexibility.

### Statistical analysis

To determine the prevalence of NSLBP, data were analysed using descriptive statistics using Statistical Package for the Social Sciences (SSPS). Chi-square tests were employed to investigate the variations in NSLBP prevalence particular to each sport. Statistical significance was set at p<0.05. Descriptive statistics and chi-square tests were used to explore associations between NSLBP, sport type, scapular dyskinesia, and latissimus dorsi flexibility.

## Results

### Participants characteristics

The study involved 52 amateur overhead athletes with a mean age of 22. The participants included 27 males and 25 females. The distribution of participants by sport was as follows: swimming (5), tennis (5), basketball (10), netball (10), soccer goalkeeping (7), and volleyball (15). The average training sessions was four training sessions per week.

### NSLBP prevalence

Twenty-five percent of the subjects had NSLBP. There was no discernible variation in the prevalence of NSLBP based on gender (males: 26%, females: 24%). There was variation in the degree of NSLBP; 12% of participants reported mild pain, 8% moderate pain, and 5% severe pain that interfered with their ability to perform in sports. These levels of pain severity were self-reported by participants using Keele STarT Back Screening Tool.

### Sport-specific prevalence

Compared to players in other sports, volleyball and basketball players had higher prevalence rates of NSLBP (33% and 30%, respectively). The prevalence rate for netball players was 20%, whereas the rates for swimmers, tennis players, and soccer goalkeepers were 10%, 12%, and 14%, respectively (Table 1).

### Grades of scapular dyskinesia

Scapular dyskinesia was present in 48% of the participants. The grades were distributed as follows: Grade 1 (20%), Grade 2 (25%), and Grade 3 (13%) (Table 2). Some participants presented with different grades in each shoulder, which may explain the total percentage exceeding 48%. There were no significant differences in scapular dyskinesia grades between males and females (Table 2).

### Latissimus dorsi flexibility

Forty percent of the subjects showed reduced latissimus dorsi muscular flexibility. A statistically significant correlation was seen between the occurrence of scapular dyskinesia and impaired latissimus dorsi flexibility (p<0.05) (Table 3).

## Discussion

### Prevalence of NSLBP

The 25% prevalence of NSLBP in this study is consistent with findings from other research on athletes. For instance, a study reported a prevalence of 32% among athletes engaged in sports involving repetitive spinal loading.^[1]^ The prevalence of non-specific low back pain (NSLBP) among professional overhead athletes has been a significant concern, with studies indicating a substantial burden of this condition within this population, highlighting a higher point prevalence of low back pain in athletes compared to the general population.^[1,12,13]^ This emphasises the elevated risk of NSLBP in athletes, particularly those engaged in overhead activities.^[14]^ Another study sheds light on the increased prevalence of back pain in elite athletes exposed to repetitive overhead activities. This suggests that specific mechanisms related to these movements may contribute to the higher prevalence of NSLBP in this athletic group.^[1]^ Moreover, it was found that even recreational athletes who were not part of the present study had a prevalence rate of 28%, suggesting that the risk extends beyond professional athletes.^[15]^

### Sport-specific prevalence

The higher prevalence rates of non-specific low back pain (NSLBP) in volleyball (33%) and basketball (30%) players can be attributed to the intense and repetitive nature of these sports. Volleyball players frequently perform overhead serves and spikes, which impose cumulative stress on the lumbar spine.^[16]^ Similarly, basketball players experience repetitive jumping and shooting, contributing to similar stress. These activities involve frequent high-impact actions and repetitive spinal loading, placing significant loads on the lower back and increasing the risk of NSLBP.^[16]^

In contrast, sports such as swimming and tennis involve more balanced and repetitive movements. This may explain the lower prevalence rates of NSLBP observed in these athletes.^[17]^ The latissimus dorsi is crucial in stabilising the trunk, essential for maintaining proper posture and reducing undue stress on the lumbar spine. However, if the latissimus dorsi is excessively tight or stiff, it could disrupt trunk stability and lead to compensatory movements that stress the lumbar spine more.^[10,11]^ Our study measured the length of the latissimus dorsi to explore its relationship with NSLBP. The findings suggest that increased stiffness in this muscle may influence lumbar spine stress and overall spinal alignment. A rigid latissimus dorsi might impair the muscle’s ability to absorb shock and adapt to varying loads, potentially raising the risk of low back pain.^[18]^ The condition and elasticity of the latissimus dorsi are also closely linked to swimming performance. For example, muscle stiffness in this area can affect the efficiency of swimming strokes and overall comfort. Increased stiffness may restrict the range of motion or trunk stability, negatively impacting performance and exacerbating lumbar discomfort.^[11,19]^

Furthermore, the function of the latissimus dorsi can influence swimming technique and contribute to low back pain. Improper use of the latissimus dorsi might lead to compensatory movements, such as excessive trunk rotation or altered spinal alignment, placing additional stress on the lumbar region.^[19]^ Focusing on proper technique and muscle conditioning is essential to mitigate these risks. Incorporating flexibility and strengthening exercises for the latissimus dorsi can help ensure balanced muscle function and reduce the risk of low back pain.

### Gendered differences

Our study found no significant gender differences in the prevalence of NSLBP (males: 26%, females: 24%). This contrasts with a study that reported higher rates of NSLBP in female athlete.^[20]^ However, our findings are consistent with research showing no significant gender differences in NSLBP among professional athletes.^[21]^ The similar training regimens and physical demands placed on male and female athletes in overhead sports may contribute to this lack of difference. Additionally, a study suggests hormonal differences, muscle strength, and flexibility variations might not significantly impact NSLBP prevalence in this specific athletic population.^[20]^

### Latissimus dorsi flexibility

The significant association between reduced latissimus dorsi flexibility and scapular dyskinesia observed in this study underscores the importance of muscle flexibility for maintaining optimal scapular function. Tightness in the latissimus dorsi can restrict scapular motion, leading to compensatory movements and an increased risk of dyskinesia and NSLBP.^[4,5,11]^ This highlights the need for flexibility exercises targeting the latissimus dorsi to prevent such compensatory movements.^[10]^ Adequate muscle flexibility through stretching and flexibility exercises is crucial for avoiding lower back strain in overhead athletes. Incorporating both flexibility and strength training can improve scapular kinematics and reduce the risk of musculoskeletal injuries.^[19]^

When an overhead athlete has a shortened latissimus dorsi, it restricts shoulder flexion and abduction. This necessitates compensatory movements in the thoracic and lumbar spine, potentially leading to hyperextension of the thoracic spine to achieve the required range of motion. This hyperextension increases lumbar lordosis, a condition characterised by excessive inward curvature of the lower back. Such altered spinal alignment stresses the lumbar spine and its supporting muscles, leading to muscle fatigue and strain.^[10,19]^ Additionally, the latissimus dorsi’s attachment to the iliac crest can induce an anterior pelvic tilt, further exacerbating lumbar lordosis. As part of the posterior oblique sling—a myofascial system crucial for stabilising the pelvis and spine during movement—tightness in the latissimus dorsi disrupts this function.^[22]^ This disruption causes further compensatory adjustments and increases the risk of low back pain. Addressing latissimus dorsi flexibility through targeted exercises can restore proper shoulder and spinal mechanics, reducing compensatory strain and preventing low back pain in overhead athletes. Studies support these biomechanical and anatomical interconnections, emphasising the importance of maintaining muscle flexibility and proper kinetic chain function to prevent injury.^[10,22]^

### Clinical implications

The findings of this study suggest that targeted interventions addressing muscle flexibility and scapular function could effectively reduce the prevalence of NSLBP among overhead athletes. Rehabilitation programs focusing on improving scapular stability and flexibility of the latissimus dorsi muscle may help prevent and manage NSLBP. Additionally, sport-specific training programs that incorporate exercises to strengthen the core and enhance overall body mechanics should be emphasised. Coaches and trainers should be aware of the high prevalence of NSLBP in certain sports and implement preventive measures accordingly. Systematic reviews have shown that multi-faceted intervention programs, including education, strength training, and flexibility exercises, are most effective in preventing and managing NSLBP in athletes.^[15]^

Preventive strategies should focus on addressing the specific biomechanical demands of each sport; for volleyball and basketball players, exercises that improve shoulder stability and reduce repetitive stress on the lower back are essential. In line with the findings, volleyball players should incorporate exercises that enhance core stability and reduce lumbar spine load.^[22,23]^ Maintaining overall muscle flexibility and strength is crucial for athletes in other sports. Screening programs to identify athletes at risk of NSLBP and implementing early intervention strategies can also help reduce the incidence of this condition. Physiotherapists or sports personnel should thoroughly screen, including tests such as the latissimus dorsi length and scapular dyskinesia tests.

Incorporating educational sessions on proper technique and body mechanics can further prevent NSLBP. This study supports the inclusion of scapular control and muscle flexibility in preventative strategies for NSLBP among overhead athletes.

### Future research

Future research should focus on several key areas to enhance our understanding and management of NSLBP among overhead athletes. First, it is essential to explore the effectiveness of specific interventions in reducing NSLBP prevalence. Longitudinal studies would provide valuable insights into how training modifications and rehabilitation programs impact NSLBP. Additionally, investigating the role of psychological stress and training load could offer a further understanding of their contributions to NSLBP. Research into biomechanical load distribution during overhead and non-overhead movements is crucial for identifying stressors contributing to NSLBP. Future studies should also consider the effects of non-overhead movements on lumbar spine stress, the role of neuromuscular control in managing biomechanical loads, and its influence on NSLBP.

Comparing the impact of overhead versus non-overhead sports on lumbar spine health could help identify specific risk factors and inform targeted preventive measures. Finally, evaluating the efficacy of various interventions, including flexibility and strength training, will provide insights into the most effective strategies for managing and preventing NSLBP. Future research can contribute to a more comprehensive understanding of NSLBP and improve prevention and treatment approaches by addressing these areas.

### Limitations

While this study provides valuable insights into the prevalence and risk factors of non-specific low back pain (NSLBP) among amateur overhead athletes, several limitations should be considered:

#### Sample size and generalizability

Although sufficient for preliminary analysis, the sample size of 52 participants may limit the findings’ generalizability. A larger sample size could provide more robust and representative data. Additionally, the study’s focus on a single tertiary institution in Gauteng Province may not fully represent amateur overhead athletes across South Africa or other regions, potentially limiting the applicability of the results to broader populations.

#### Sport-specific bias

The distribution of participants across different sports was uneven, with more volleyball and basketball players than other sports. This imbalance may affect the accuracy of sport-specific prevalence rates and introduce bias in the findings. Future studies should aim for a more balanced representation of various sports to improve the generalizability of the results.

#### Methodology limitations

The study relied on self-reported data for the prevalence and impact of NSLBP, which could be subject to reporting bias or inaccuracies. Participants’ perceptions of pain and its impact may differ from clinical assessments or objective measures. Incorporating objective diagnostic tools and clinical evaluations could enhance the reliability of the findings.

#### Measurement variability

Despite efforts to standardise test procedures and measurement techniques, variability in how different testers performed tests may have influenced the results. Although training sessions were conducted and standard operating procedures established, the potential for measurement inconsistencies remains a limitation.

#### Cross-sectional design

The study’s cross-sectional design provides a snapshot of the prevalence and risk factors simultaneously. However, this design does not account for changes in NSLBP over time or the effects of intervention. Longitudinal studies would be beneficial for understanding the progression of NSLBP and the impact of preventive or therapeutic measures.

#### Potential confounding factors

Other factors such as lifestyle, previous injuries, and physical conditioning may also influence the development of NSLBP but were not fully explored in this study. Future research should consider these additional variables to provide a more comprehensive understanding of the risk factors associated with NSLBP.

#### Bias in recruitment

The recruitment of participants through university sports clubs and notices may have introduced selection bias, as individuals who are more engaged or have specific health concerns might be overrepresented. A more diverse recruitment strategy could help mitigate this bias. While this study offers important insights into the prevalence and risk factors of NSLBP among amateur overhead athletes, addressing these limitations in future research will be crucial for developing more accurate and generalisable findings.

## Conclusion

This study highlights a substantial prevalence of non-specific low back pain (NSLBP) among amateur overhead athletes, with variations observed across different sports. The findings emphasise the importance of addressing muscle flexibility and scapular function as part of targeted prevention and treatment strategies. Our results indicate a strong association between reduced flexibility of the latissimus dorsi and increased risk of NSLBP. However, it is crucial to recognise that while these associations are significant, they do not establish causation.

To advance our understanding, future research should explore causal relationships by investigating the impact of specific interventions on NSLBP prevalence and severity. Additionally, examining other factors, such as psychological stress and training load, along with the role of biomechanical load distribution and neuromuscular control, will provide deeper insights into the multifactorial nature of NSLBP. Developing and implementing sport-specific strategies based on these insights will be essential for effectively reducing the impact of NSLBP on athletes.

## Figures and Tables

**Table 1:** Prevalence of non-specific low back pain (NSLBP) and scapular dyskinesia by sport

Sport	Number of participants	Prevalence of NSLBP (%)	Prevalence of scapular dyskinesia (%)

Volleyball	15	33	60
Basketball	10	30	50
Netball	10	20	55
Soccer Goalkeeping	7	14	57
Tennis	5	12	40
Swimming	5	10	60

**Table 2:** Scapular dyskinesia grades distribution

Grade	Number of participants	Percentage (%)

1	10	20
2	13	25
3	7	13

**Table 3:** Association between Latissimus Dorsi Flexibility and Scapular Dyskinesia

Latissimus Dorsi Flexibility	Number of participants	Presence of Scapular Dyskinesia (%)

Normal	31	42
Reduced	21	81

## References

[1] Fett D, Trompeter K, Platen P (2019). Prevalence of back pain in a group of elite athletes exposed to repetitive overhead activity. PLoS One.

[2] Abd Rahman NA, Li S, Schmid S, Shaharudin S (2023). Biomechanical factors associated with non-specific low back pain in adults: A systematic review. Phys Ther Sport.

[3] Ellenbecker TS, Aoki R (2020). Step by step guide to understanding the kinetic chain concept in the overhead athlete. Curr Rev Musculoskelet Med.

[4] Sciascia A, Kibler WB (2022). Current views of scapular dyskinesis and its possible clinical relevance. Int J Sports Phys Ther.

[5] Taghizadeh S, Pirouzi S, Hemmati L, Khaledi F, Sadat A (2017). Clinical evaluation of scapular positioning in patients with nonspecific chronic low back pain: a case-control study. J Chiropr Med.

[6] Schneider AJ, Butcher RB (1993). For the love of the game: A philosophical defense of amateurism.

[7] Robinson HS, Dagfinrud H (2017). Reliability and screening ability of the StarT Back screening tool in patients with low back pain in physiotherapy practice, a cohort study. BMC Musculoskelet Disord.

[8] Kibler WB (1998). The role of the scapula in athletic shoulder function. Am J Sports Med.

[9] McClure P, Tate AR, Kareha S, Irwin D, Zlupko E (2009). A clinical method for identifying scapular dyskinesis, part 1 reliability. J Athl Train.

[10] Dawood M, Bekker PJ, van Rooijen AJ, Korkie E (2018). Inter- and intra-rater reliability of a technique assessing the length of the latissimus dorsi muscle. S Afr J Physio.

[11] Herrington LC, Horsley IG (2014). Effects of latissimus dorsi length on shoulder flexion in canoeists, swimmers, rugby players, and controls. J Sport Health Sci.

[12] Fett D, Trompeter K, Platen P (2017). Back pain in elite sports: A cross-sectional study on 1114 athletes. PLoS One.

[13] Noormohammadpour P, Rostami M, Mansournia MA, Farahbakhsh F, Pourgharib Shahi MH, Kordi R (2016). Low back pain status of female university students in relation to different sport activities. Eur Spine J.

[14] Ławnicki J, Hadała M, Zaręba W (2017). Low back pain in the overhead athletes: Evaluation and treatment based on movement system. Pol Ann Med.

[15] Farahbakhsh F, Rostami M, Noormohammadpour P (2018). Prevalence of low back pain among athletes: A systematic. J Back Musculoskelet Rehabil.

[16] Yabe Y, Hagiwara Y, Sekiguchi T (2020). Association between lower back pain and lower extremity pain among young volleyball players: A cross-sectional study. Phys Ther Sport.

[17] Zhao K, Hohmann A, Faber I, Chang Y, Gao B (2020). A 2-year longitudinal follow-up of performance characteristics in Chinese male elite youth athletes from swimming and racket sports. PLoS One.

[18] Kibler W, Sciascia A (2019). (The Role of the Scapula in the Overhead Athlete. Mechanics, Pathomechanics and Injury in the Overhead Athlete.

[19] Feijen S, Tate A, Kuppens K, Struyf T, Claes A, Struyf F (2020). Intrarater and interrater reliability of a passive shoulder flexion range of motion measurement for latissimus dorsi flexibility in young competitive swimmers. J Sport Rehabil.

[20] Kikuchi R, Hirano T, Watanabe K (2019). Gender differences in the prevalence of low back pain associated with sports activities in children and adolescents: a six-year annual survey of a birth cohort in Niigata City, Japan. BMC Musculoskelet Disord.

[21] Mortazavi J, Zebardast J, Mirzashahi B (2015). Low back pain in athletes. Asian J Sports Med.

[22] Singh A, Patel M, Sharma M (2023). Influence of muscle activation of posterior oblique sling in different hip positions among three different shoulder movements in overhead athletes: an observational study. Adv Rehabil.

[23] Dal Farra F, Arippa F, Carta G, Segreto M, Porcu E, Monticone M (2022). Sport and non-specific low back pain in athletes: a scoping review. BMC Sports Sci Med Rehabil.

[24] Jones SD, Safran MR (2024). Current concepts: the hip, core, and kinetic chain in the overhead athlete. J Shoulder Elbow Surg.

